# Formaldehyde initiates memory and motor impairments under weightlessness condition

**DOI:** 10.1038/s41526-024-00441-0

**Published:** 2024-10-28

**Authors:** Tianhao Mei, Ying Chen, Yajuan Gao, Hang Zhao, Xingzhou Lyu, Jing Lin, Tianye Niu, Hongbin Han, Zhiqian Tong

**Affiliations:** 1 Beijing Geriatric Hospital, Beijing, China; 2 Zhejiang Provincial Clinical Research Center for Mental Disorders, The Affiliated Wenzhou Kangning Hospital, School of Mental Health, Wenzhou Medical University, Wenzhou, Zhejiang China; 3 Department of Radiology, Peking University Third Hospital, Beijing, China. Key Laboratory of Magnetic Resonance Imaging Equipment and Technique, Beijing, China; 4 NMPA key Laboratory for Evaluation of Medical Imaging Equipment and Technique, Beijing, China; 5 Institute of Medical Technology, Peking University Health Science Center, Beijing, China; 6 Shenzhen Bay Laboratory, Shenzhen, China; 7 University of Science and Technology of China, Anhui, China

**Keywords:** Pathogenesis, Neuroscience

## Abstract

During space flight, prolonged weightlessness stress exerts a range of detrimental impacts on the physiology and psychology of astronauts. These manifestations encompass depressive symptoms, anxiety, and impairments in both short-term memory and motor functions, albeit the precise underlying mechanisms remain elusive. Recent studies have revealed that hindlimb unloading (HU) animal models, which simulate space weightlessness, exhibited a disorder in memory and motor function associated with endogenous formaldehyde (FA) accumulation in the hippocampus and cerebellum, disruption of brain extracellular space (ECS), and blockage of interstitial fluid (ISF) drainage. Notably, the impairment of the blood-brain barrier (BBB) caused by space weightlessness elicits the infiltration of albumin and hemoglobin from the blood vessels into the brain ECS. However, excessive FA has the potential to form cross-links between these two proteins and amyloid-beta (Aβ), thereby obstructing ECS and inducing neuron death. Moreover, FA can inhibit N-methyl-D-aspartate (NMDA) currents by crosslinking NR1 and NR2B subunits, thus impairing memory. Additionally, FA has the ability to modulate the levels of certain microRNAs (miRNAs) such as miRNA-29b, which can affect the expression of aquaporin-4 (AQP4) so as to regulate ECS structure and ISF drainage. Especially, the accumulation of FA may inactivate the ataxia telangiectasia-mutated (ATM) protein kinase by forming cross-linking, a process that is associated with ataxia. Hence, this review presents that weightlessness stress-derived FA may potentially serve as a crucial catalyst in the deterioration of memory and motor abilities in the context of microgravity.

## Introduction

The condition of weightlessness is a unique setting encountered by individuals when they venture into space. In recent times, owing to the swift progress of the global space sector, the forthcoming establishment of advanced space stations will furnish astronauts with a cutting-edge platform to engage in extended space missions. Consequently, ensuring the long-term well-being of astronauts in space has emerged as a central area of concern in contemporary aerospace medicine research. Over the course of millions of years, human beings have developed physiological structures and functional characteristics that are well-suited to the gravitational conditions of the Earth^1^. However, extended periods of weightlessness have been found to have inevitable physiological and psychological effects on the human body^2^. Extensive research in aerospace medicine has demonstrated that weightlessness not only significantly impacts the physical well-being of astronauts, but also represents a crucial factor in the development of brain damage among them^3^. The aforementioned phenomenon not only hampers the astronauts’ equilibrium performance, motor control, and short-term memory storage capabilities, but also exhibits a high susceptibility to instigating neurological disorders, encompassing cognitive functions such as reaction, judgment, decision-making, and other cognitive processes. Consequently, this significantly impairs the astronauts’ operational efficacy.

Notably, the primordial gaseous molecules in the course of early evolution, such as: formaldehyde (FA), carbon monoxide (CO), nitric oxide (NO), and hydrogen sulfide (H_2_S), exist in the brain and are considered to act as gaseous neuromodulators to regulate brain functions^4,5^. Endogenous and active FA is mainly derived from the demethylation of sarcosine (SA), methylamine (MMA), DNA or histone via mitochondrial sarcosine dehydrogenase (SARDH), semicarbazide-sensitive amine oxidase (SSAO) and demethylase^6^. Surprisingly, certain external stimuli such as hindlimb unloading (HU) simulating microgravity, spatial learning, and electrical stimulation, have been found to elicit the generation of FA in the brain through the involvement of SSAO and SARDH^4,7^. Additionally, other stress also contributes to the endogenous production of FA^8,9^. It has been found that stress can induce the accumulation of FA in the brain; especially, microgravity stress leads to an increase in FA content in the brains of the HU models mimicking the astronauts in space^4,7^. However, when FA reaches a certain concentration, it does not continue to rise, but remains at a certain pathological concentration^7^. After astronauts return to Earth, the microgravity stress disappears, the activity and expression contents of formaldehyde dehydrogenase (FDH) in the brain may return to normal levels^10^, thereby degrading excessive FA in the brain^11^.

In recent years, the advancement of magnetic resonance imaging (MRI) technology has facilitated the detection of water diffusion in the brain extracellular space (ECS) through the utilization of tracers^12^. A high-resolution MRI technique for aquaporin-4 (AQP4) in vivo has been established, which greatly improves the sensitivity of MRI measurement of water molecule transmembrane transport by specifically labeling and amplifying its magnetic resonance signal^13^. Consequently, this technique has become the preferred method for investigating the microstructural characteristics of neural tissue. Notably, the glymphatic system removes brain interstitial solutes, with AQP4 being a key component^14^. AQP4’s involvement in the exchange of cerebrospinal fluid (CSF) and interstitial fluid (ISF) has led to hypotheses about the impact of the excessive FA on AQP4 and the glymphatic system’s function.

MicroRNAs (miRNAs) are a notable group of endogenous non-coding single-stranded RNAs that have been observed to play a significant role in various neural functions. Many studies have confirmed that FA can regulate the levels of miRNAs^15–17^. The use of drug delivery systems that target the lymphatic system and brain ECS to remove accumulated endogenous FA and regulate levels of microRNAs is crucial for maintaining the cognitive health of astronauts and ensuring the success of future space exploration missions. Hence, this review presents a comprehensive examination of the advancements in research pertaining to the possible molecular mechanisms underlying memory and motor dysfunctions induced by FA derived from space weightlessness, as elucidated in the preceding relevant literatures.

## Role of CSF-ISF exchange in maintaining brain functions

The brain ECS is a non-uniform spatial arrangement situated adjacent to the neural network, measuring ~38–64 nm in width. It encompasses 15-20% of the brain’s total volume, surpassing the previously emphasized cerebral blood vessel space (3-5%)^18^. The ECS wall structure comprises the cell membranes of neurons, astrocytes, oligodendrocytes, microglia, and other cellular components, in addition to the cerebral vascular wall. The brain ECS comprises ISF, extracellular matrix (ECM), and a variety of essential nutrients, ions, and neurotransmitters necessary for the sustenance and operation of nerve cells^19^. Within the brain ECS, ISF facilitates the transportation of neurotransmitters and nutrients to nerve cells, as well as the exchange of metabolic waste with the parenchyma^20^. Consequently, ISF plays a crucial role as a mediator for nutrient provision, waste elimination, and intercellular communication within brain tissue. The primary physiological role of the human lymphatic system is the elimination of metabolites from the body. Lymphatic vessels are distributed extensively throughout the body, contrary to the previous belief that the lymphatic system was absent in the brain^21^. Historically, it was thought that metabolites in the brain were primarily eliminated through CSF circulation. The CSF, a transparent and colorless fluid, fills the ventricles of the brain and the subarachnoid space, serving to safeguard the central nervous system against external shocks and maintain its normal metabolic functions^22^. Through the progression of scientific inquiry, researchers have discovered that the CSF circulation, in isolation, is insufficient for the expeditious elimination of the majority of metabolites. Consequently, they embarked upon an investigation to ascertain the existence of alternative pathways for metabolite removal within the brain.

In 2012, it was found that there is the existence of a CSF-ISF convective system based on the perivascular space in the mouse brain by using the method of two-photon in-vivo imaging to explore the dynamic flow process of CSF in the mouse brain^23^, further refining the mechanism of waste removal in the nervous system^24^. This convective system employs AQP4 on the terminal feet of astrocytes to facilitate the transportation of amyloid-beta (Aβ) protein and metabolites to the CSF for clearance. It is referred to as the glymphatic system due to its functional similarity to the peripheral lymphatic system^25^. However, there is an observed decline in the functionality of the brain’s glymphatic system in various disease states, including traumatic brain injury, Alzheimer’s disease (AD) and Parkinson’s disease (PD)^26–28^. This suggests that the proper functioning of the glymphatic system is crucial for maintaining the brain’s homeostasis.

**Fig. 1:**
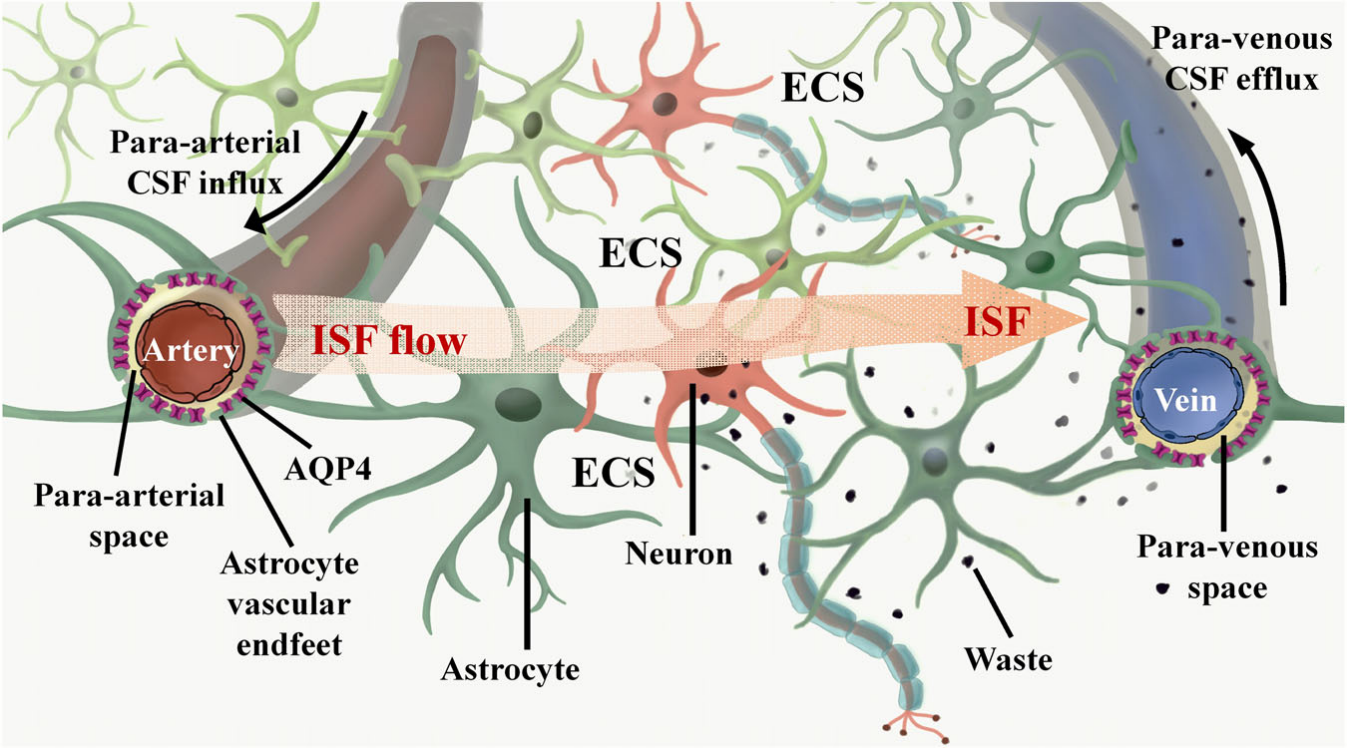
Schematic diagram of the exchange mechanism between the ISF and CSF in the brain ECS. In brief, the ISF within the brain ECS undergoes an exchange with the CSF, akin to the process observed between tissue fluid and plasma. This exchange occurs as the CSF enters the brain parenchyma via the PVS along the cerebral surface arteries and perforating arterioles. Subsequently, the CSF interacts with the ISF within the cerebral ECS, and ultimately, the CSF exits the brain by flowing into the venous PVS. AQP4 aquaporin-4, CSF cerebrospinal fluid, ECS extracellular space, ISF interstitial fluid, PVS perivascular space.

In brain tissue, ISF can undergo rapid transportation through the active or passive involvement of AQP4 located on the astrocyte end-foot. This transportation process facilitates the translocation of ISF from the end of the astrocyte peduncle to the perivascular space. Subsequently, the ISF is exchanged with the CSF and distributed to various locations^29,30^, ultimately draining into the peripheral lymphatic system^31,32^. Consequently, the intermingling and movement of the ISF and CSF within the brain tissue establish a connection with the peripheral lymphatic system, which is widely acknowledged as the primary pathway for substance exchange and waste elimination in the brain tissue^33^. Explorations into brain ECS and glymphatic system of the brain have yielded fresh insights into the underlying pathological mechanisms of brain disorders and hold promise as prospective therapeutic targets for a range of brain diseases (Fig. 1).

## Effects of microgravity-derived fa on cognitive functions

Gaseous FA, a colorless and volatile gas with an irritating odor, is widely recognized for its use as a preservative^34^. It is noteworthy that primary gaseous FA has been regarded as the earliest and simplest form to emerge during the early stages of Earth’s evolution, encompassing carbon, hydrogen, and oxygen elements within a small organic molecule^35^. In fact, FA serves as the primary precursor for numerous intricate organic compounds, such as amino acids, RNA, DNA, and proteins^36^. It is widely recognized that FA is a well-established indoor air pollutant that has been observed to cause memory deficits in animals and cognitive decline in humans^37,38^. Interestingly, FA is found in all vertebrate cells, potentially as a byproduct of various metabolic reactions such as methanol oxidation, DNA or histone demethylation^39^. Recent research has demonstrated that endogenous FA is present in the cytoplasm, nucleus, and subcellular organelles of all organisms, and it plays a role as a gas signaling molecule in the processes of learning and memory^39,40^.

### Metabolism of FA in the living organism

**Fig. 2:**
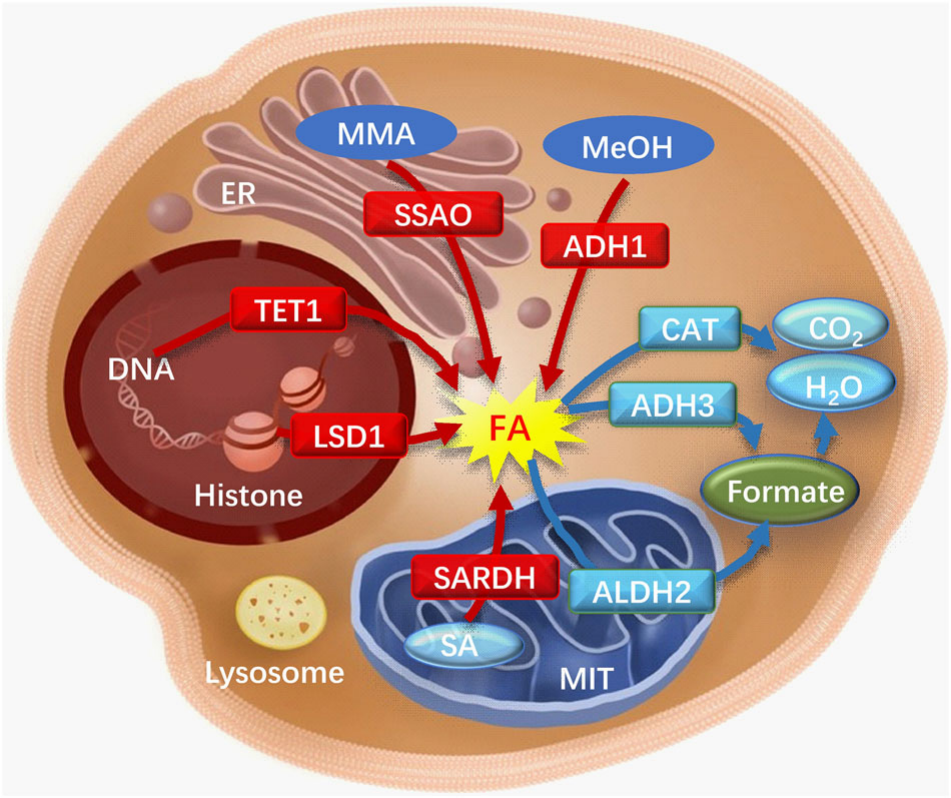
Multiple metabolic pathways of endogenous FA in the cells. Red arrows: FA-generating pathways; blue arrows: FA-degrading pathways. ADH1 alcohol dehydrogenase 1, ADH3 alcohol dehydrogenase 3, ALDH2 aldehyde dehydrogenase 2, CAT catalase, ER endoplasmic reticulum, FA formaldehyde, LSD1 lysine special demethylase 1, MeOH methanol, MMA monomethylamine, MIT mitochondria, SA sarcosine, SARDH sarcosine dehydrogenase, SSAO semicarbazide-sensitive amine oxidase, TET1 TET methylcytosine dioxygenase 1.

Unexpectedly, apart from the inhalation of exogenous FA, the human body also synthesizes endogenous FA via diverse pathways, with enzyme-catalyzed reactions serving as the primary mechanism. For instance, the breakdown of endogenous amine substances, such as methylamine, histamine, and polyamines, into FA is facilitated by SSAO. Additionally, FA is generated through DNA demethylation catalyzed by lysine demethylase (LSD), and mitochondrial cytochrome P450 enzymes oxidize exogenous compounds to produce FA (Fig. 2). Research has demonstrated that the stress-induced activation of SSAO, which is widely distributed in vascular cells, smooth muscle cells, and adipocytes, facilitates the deamination of various exogenous and endogenous monoamines^9^. This enzymatic activity leads to the production and accumulation of endogenous FA^41^. Numerous investigations have indicated that FA can disrupt the structural integrity of proteins, thereby impacting the physiological function of cells. Prolonged exposure to FA has been associated with detrimental effects on multiple human systems, including the respiratory, digestive, immune, and neurological systems^42^.

### Microgravity stress induces FA accumulation in the brains

**Fig. 3:**
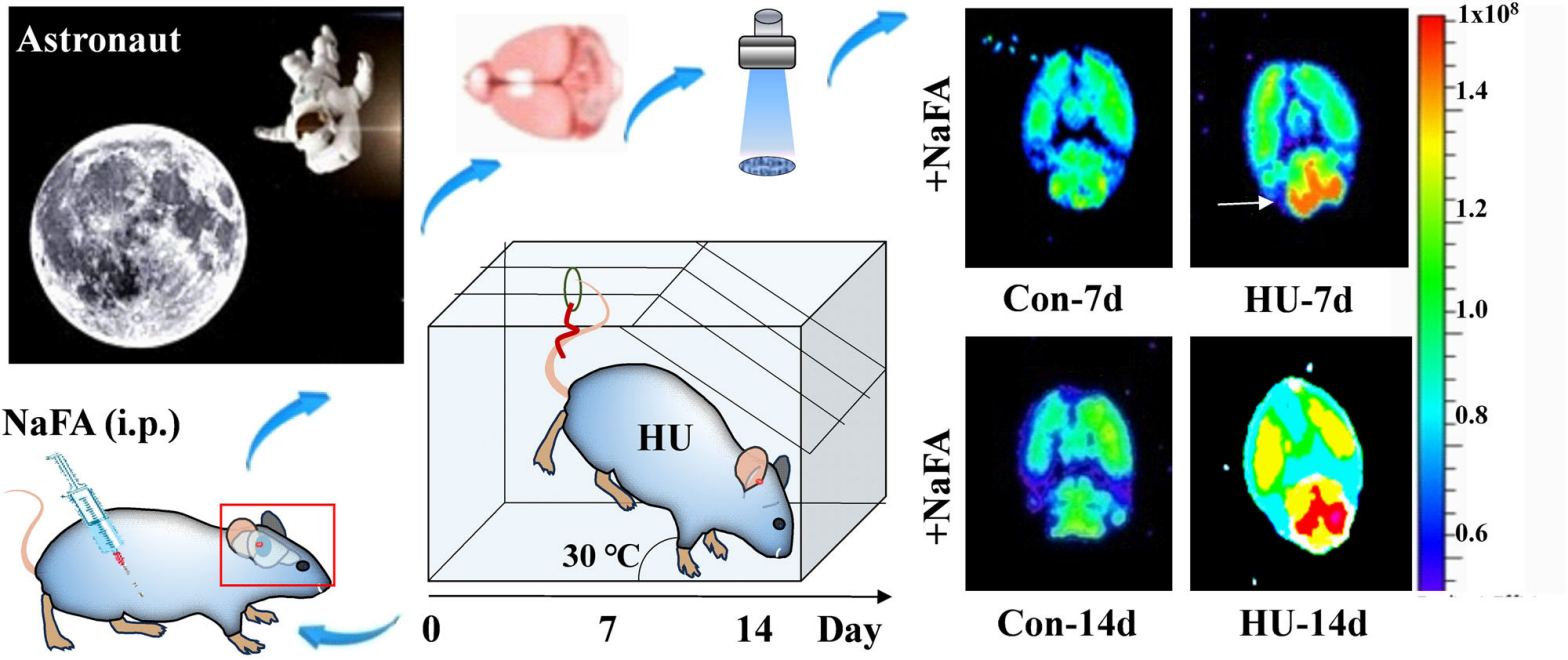
Abnormal high levels of FA in the hippocampus and cerebellum in the mouse model of hindlimb unloading which simulates weightlessness by using tail suspension to unload the hind limbs. Schematic overview of an i*n-vivo* animal imaging system with a NaFA probe was used to image brain FA in a mouse model of unloading the hind limbs for consecutive 2 weeks (*λ*_ex\em_ = 440/550 nm). Reproduced with the permission of ref. ^7^, copyright@Communications Biology, 2021. FA formaldehyde, HU hindlimb unloading, i.p. intraperitoneal injection, NaFA a free FA fluorescence probe, Con-7d feed for 7 days in normal conditions, HU-7d feed for 7 days in hindlimb unloading conditions, Con-14d feed for 14 days in normal conditions, HU-14d feed for 14 days in hindlimb unloading conditions.

Microgravity is a significant stressor that can induce prolonged stress in astronauts. For example, the simulation of space weightlessness through HU for a continuous period of 2 weeks has been found to trigger the production and accumulation of FA in the hippocampus and cerebellum of mice models. This effect is achieved through the activation of both the SSAO and SARDH^4,7^ (Fig. 3). During this period, significant sympathetic neurotransmitters, including adrenaline, experience a surge^43^, and adrenaline, facilitated by monoamine oxidase-A (MAO-A), undergoes deamination resulting in the production of hydrogen peroxide (H_2_O_2_) and MMA^44^. MMA is readily metabolized by SSAO, leading to the generation of FA^45,46^. Concurrently, the metabolic generation of H_2_O_2_ can directly inhibit the function of FDH^10^, thereby impeding the timely degradation of FA^11^. Consequently, this impediment results in the excessive buildup of endogenous FA within brain tissues, subsequently instigating a pronounced state of oxidative stress and the generation of a substantial quantity of free radicals and other detrimental compounds. Excessive free radicals in brain tissues, facilitated by the metal ion catalytic system, initiate an assault on the amino or imino groups of amino acid molecules^47–49^. This process results in the conversion of amino acids into carbonyl derivatives, thereby causing structural disruption and functional impairment of protein molecules. Consequently, neuronal cells experience structural damage, ultimately culminating in apoptosis or death. These detrimental effects on neuronal cells can manifest as symptoms including depression, anxiety, and memory disorders^50–52^.

### Microgravity stress damages ECS structure and ISF drainage

The hippocampus, situated between the thalamus and the medial temporal lobe, is an integral component of the limbic system in the brain, primarily implicated in cognitive processes such as learning and memory^53^. Presently, it is widely accepted that the hippocampus primarily serves the purposes of short-term memory consolidation and spatial orientation. Microgravity can disrupt the typical morphology of hippocampal neurons^54^, concurrently diminishing the levels of nerve growth factor and brain derived neurotrophic factor (BDNF)^55^, thereby impeding the customary development and metabolic processes of hippocampal neurons^56^. BDNF, as a constituent of the neurotrophic factor family^57^, plays a pivotal role in the formation of both short-term and long-term memory^58^. Evidence consistent with this is that application of FA solution can downregulate BDNF^49^.

**Fig. 4:**
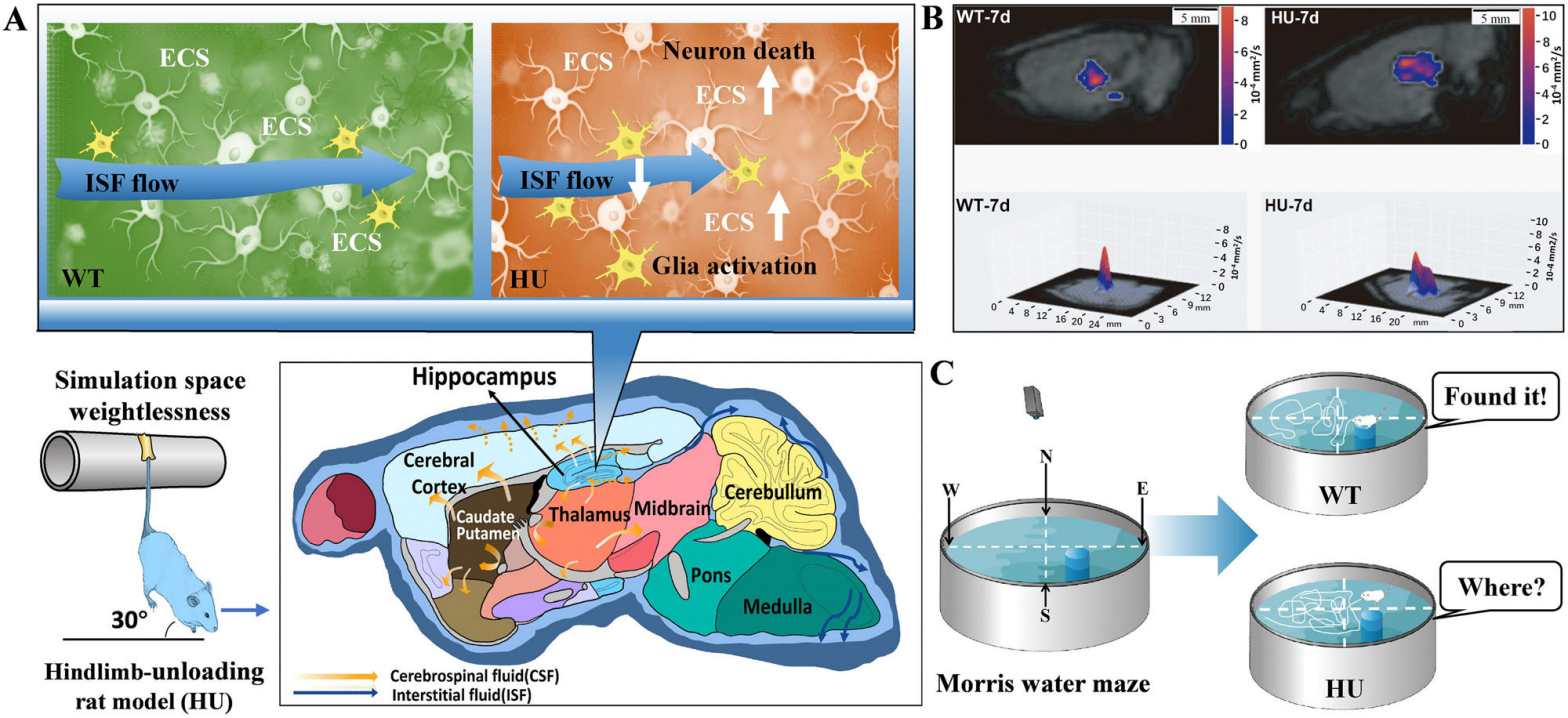
Alteration in the tortuosity of the ECS in hippocampus and the ability of spatial memory in the rat model of hindlimb unloading. **A**, **B** HU model rat exhibited an increase in the tortuosity of hippocampal ECS and a decline in the velocity of ISF drainage the diffusion associated with the activated microglia and neuron death. Reproduced with the permission of ref. ^59^, copyright@ Science China Life Sciences, 2022. **C** Decline in the ability of spatial memory in the HU model rats than the WT rats. Reproduced with the permission of ref. ^51^, copyright@Alzheimer’s & Dementia (New York, N. Y.), 2019. CSF cerebrospinal fluid, ECS extracellular space, HU rat model of hindlimb unloading, ISF interstitial fluid, WT wild-type rats.

Notably, microgravity in space modifies the convoluted nature of hippocampal ECS, resulting in disrupted ISF drainage and impaired neuromelanin sheaths. These alterations ultimately culminate in the degeneration and demise of hippocampal neurons^59^. For example, the utilization of tracer-based MRI techniques revealed notable hippocampal impairment subsequent to the simulation of microgravity conditions for a duration of 7 days in the HU model mice. This impairment was characterized by a pronounced deceleration in ISF drainage within hippocampal ECS, a reduction in diffusion rate, and a simultaneous modification in the tortuosity of the ECS. Notably, such alterations are typically irreversible^59^ (Fig. 4).

The occurrence of disturbed drainage of ISF in the hippocampal ECS may lead to the accumulation of metabolic wastes that cannot be excreted, and the occurrence of strong oxidative stress in neuronal cells, which may influence the expression of relevant proteins and trigger neuronal damage and death^54^. For example, HU simulated microgravity for 7 days can cause death of hippocampal neurons in rats, which indeed result in memory impairments^60^. Unsurprisingly, the changes in the HU rat hippocampal CA1 region have been observed after 14-days tail suspension, because the mean area of the neurons, synaptic gaps, and length of neuronal active zones in the hippocampus were markedly reduced^61^.

### FA impairs memory by suppressing NMDA receptors activity

Using the HU rat model, it has been observed that the formation of the SNARE complex, which is linked to cognitive processes such as learning and memory, is partially hindered in a simulated microgravity environment for a continuous period of 21 days^62^. Furthermore, the loss of β-synuclein was also observed in hippocampus from mice kept in simulated microgravity environment for 7 days, which serves as a molecular chaperone and effectively prevents abnormal protein aggregation^54,63^. These findings provide evidence to support the view that microgravity triggers abnormal protein aggregation in hippocampal neurons, thereby affecting the functions of learning and memory^54^.

**Fig. 5:**
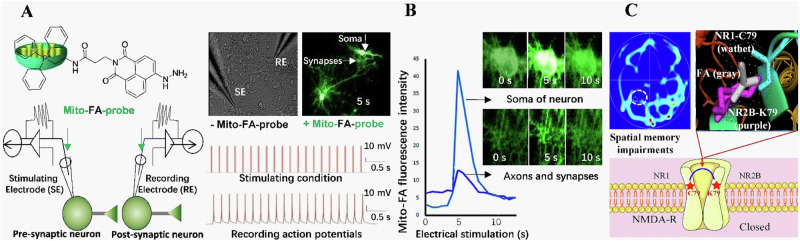
Electrical stimulation induces FA generation while excessive FA inhibits NMDA currents. **A** A train of 20 pulses in the presynaptic neurons induced multiple action potentials in the postsynaptic neurons with intracellular infusion of the mitochondria-targeting FA probe (mito-FA-probe). **B** FA generation and transduction in the axon, synapse and soma in the cultured neurons imaged by using the mito-FA probe. **C** Model of excessive FA-suppressed NMDA currents by cross-linking C79 of NR1 to K79 of NR2B. Reproduced with the permission of ref. ^4^, copyright@ Communications Biology, 2019. ATD amino-terminal domain, C cysteine, FA formaldehyde, RE recording electrode, s second, SE stimulating electrode, N N-termini of amino acid.

Although electric stimuli can elicit an elevation in the levels of endogenous FA (0.05 mM) and enhance memory formation, injection of FA at a concentration of 450 μM can impair spatial memory in healthy mice^64,65^, it is possible that excessive FA may block N-methyl-D-aspartate receptors (NMDAR). Meanwhile, it was observed that the level of FA in the cerebellum has exceeded 450 μM in the HU mouse model^7^, indicating that the level required to cause spatial memory impairment is indeed consistent with microgravity simulation. A prior investigation has indicated the possibility of intermolecular cross-linking occurring between residue C79 of NR1 and lysine (K)79 of NR2^66^, both of which are subunits of the NMDAR. However, analysis of the 3D crystal structure of NR1/NR2B (PBD ID: 4PE5) reveals that the distance between C79 and K79 is ~4 Å^67^. It is well-established that at a sufficiently high concentration, FA can act as a cross-linker for protein cysteine (C), K, and tyrosine (Y) residues^68,69^. This hypothesis posits that FA at a concentration of 450 μM could potentially hinder the functioning of the NMDAR by forming a cross-link between C79 and K79. Notably, when a single point mutation was introduced in either NR1 C79 or NR2B K79, the inhibitory effect of FA on NMDA currents in transfected CHO cells was reversed^4^. These findings provide substantial evidence supporting the notion that an excessive amount of FA can impede NMDAR activity through the cross-linking of NR1 and NR2B residues (Fig. 5).

## Effects of microgravity on motor function of astronauts

Due to the significant weakening of gravity in space, astronauts frequently encounter cephalad fluid shifting, resulting in notable changes in intracranial pressure^70^. The perception of their vestibular system is also profoundly impacted, leading to imbalance and sensations of dizziness^71^. Meanwhile, muscular and skeletal alterations have been identified as potential primary factors contributing to the decline in motor function among astronauts^72,73^. When exposed to microgravity, the musculoskeletal system lacks mechanical load, leading to muscle atrophy and bone loss, threatening the safety of long-term missions and motor function of astronauts returning to Earth^74^. However, in the HU rat model, although muscle atrophy in the hind limbs had recovered after a 2-week recovery period, the motor deficits were not reversed^75,76^. Recent studies have found that excessive FA in the muscle mainly induced gait instability but not motor disfunction^7^. Meanwhile, anatomical observations showed that the cerebellum of rats has been damaged during space flight^77^.

Astronauts usually recover from symptoms related to movement disorders and ataxia 30 days after returning to Earth^78,79^, because microgravity stress disappears and the activity and expression contents of FDH in the brain may recover to the normal levels^10^, and lead to the degradation of FA^11^. Although astronauts gradually regained their main motor function^79^, brain damage caused by changes in nanoscale neuronal structure still exists due to the acute accumulation of FA in microgravity environment^59–61^, which is one of the more likely causes of astronaut motor dysfunction. With the widespread use of MRI technology, it has been found that after astronauts return to Earth, the cerebellar structure of astronauts still undergoes changes after prolonged space flight^80^, including an increase in tissue density and gray matter volume in regions such as the cerebellar vermis^73,81^. Due to the spatial resolution of MRI typically being at the micrometer level, it is unable to display changes in the nanoscale neuronal structure caused by brain injury^82^. Hence, the precision instruments with nanometer resolution are better able to diagnose or detect space weightlessness-induced brain damage and monitor brain recovery after astronauts return to Earth.

### Cerebellar damage induces ataxia

The cerebellum serves as a crucial motor regulatory hub within the human body, primarily tasked with the maintenance of bodily equilibrium, regulation of muscle tone, and facilitation of voluntary movements. While voluntary movements are consciously initiated by the cerebral cortex, the cerebellum assumes the responsibility of coordinating these intentional actions^83^. The cerebellum integrates afferent nerve impulses of both types and modulates the movement of associated muscles via efferent fibers, thereby sustaining the coordination of voluntary movements. Consequently, cerebellar injury elicits symptoms of ataxia, characterized by impaired regulation of muscle tone and coordination of voluntary movements.

Ataxia is a motor disorder characterized by the disruption of autonomous trunk movements, resulting in the inability to maintain trunk posture and balance despite normal muscle strength. The occurrence of ataxia can be attributed to abnormalities in subcortical motor structures, including the motor cortex, basal ganglia, and cerebellum, with cerebellar ataxia being the predominant form^84^. In a manner akin to the manifestations of ataxia, astronauts encounter a progressive deterioration in their motor control and coordination throughout extended periods of space travel, subsequently presenting symptoms upon reentry to the Earth, including impaired spatial orientation during ambulation^85^, modified patterns of muscle activation^86,87^, and diminished motor coordination^88^.

### Relationship between CSF volume and ISF drainage

**Fig. 6:**
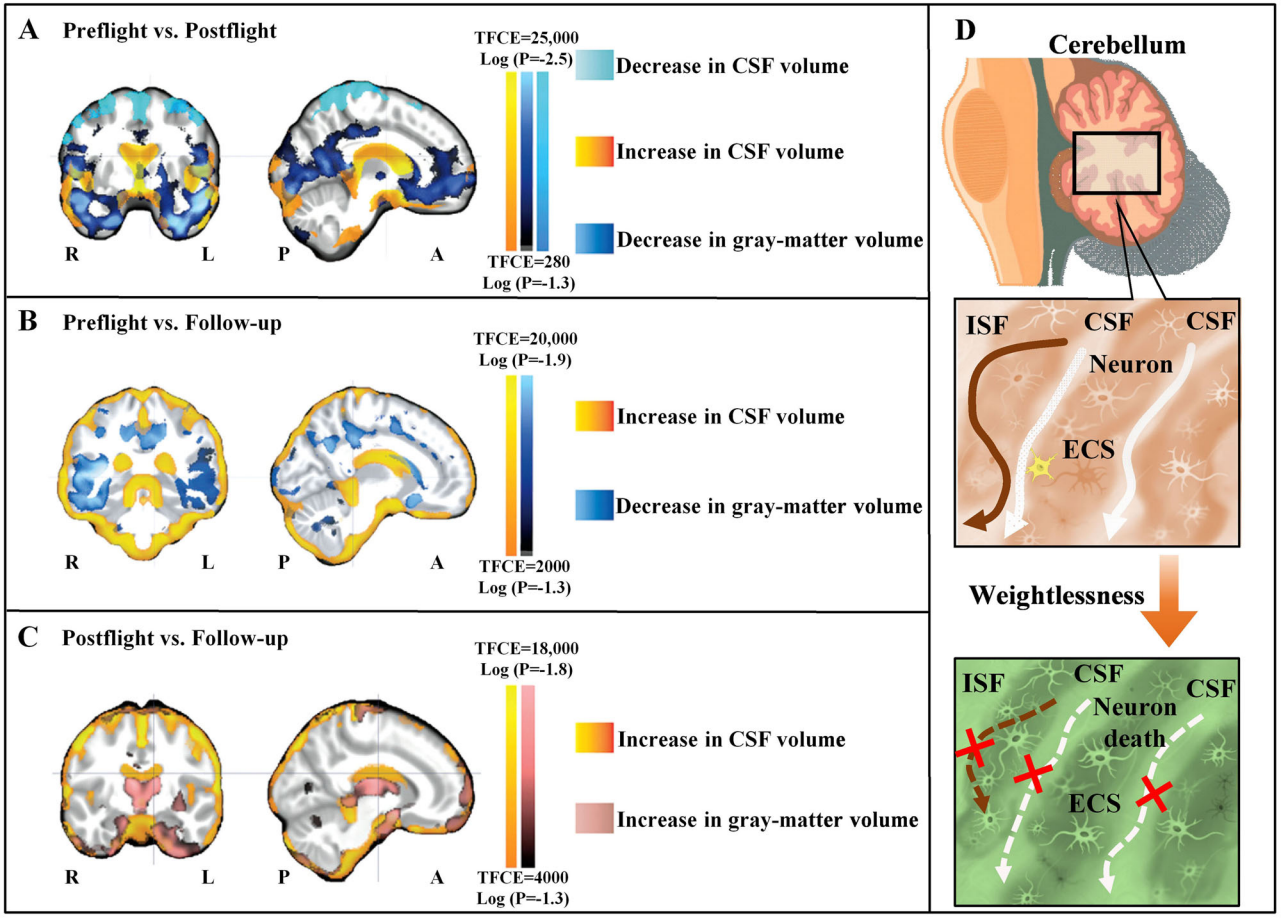
Schematic diagram of changes in the CSF volume and cerebellar ECS of astronauts before and after space flight. **A** Compared to preflight, the astronauts exhibited a decrease in the gray-matter volume (dark blue) in the orbitofrontal and temporopolar regions postflight, as well as a reduction in the CSF volume under the vertex (light blue). Conversely, the CSF volume in the cerebral ventricles and cerebellum (yellow-red) increased postflight compared to preflight. **B** Compared to preflight, there was a decrease in the gray-matter volume (dark blue) in the ventral cortical regions of the astronauts at long-term follow-up. **C** Compared to postflight, the astronauts exhibited increases in the gray-matter volume (pink) in these same regions during long-term follow-up, accompanied by residual enlargement of the subarachnoid CSF space (yellow-red) around the brain. L and R denote left and right, and A and P anterior and posterior. **D** Increase in the cerebellar CSF volume may compresses the cerebellar parenchyma, thereby reducing brain ECS volume and blocking ISF drainage for microglia activation and neuron death. Reproduced with the permission of ref. ^93^, copyright@ The New England Journal of Medicine, 2018. ISF interstitial fluid, CSF cerebrospinal fluid, ECS extracellular space, TFCE threshold-free cluster enhancement.

In recent years, research has revealed that prolonged spaceflight has led to modifications in the cerebellar structure of astronauts^89^, which plays a crucial role in regulating precise motor movements. Additionally, the vestibular system responsible for maintaining equilibrium has also been affected to a certain degree^90^. Specifically, weightlessness has been observed to disrupt the connectivity of the vestibular nuclei, resulting in diminished connectivity of the inferior cerebellar peduncle structures associated with space travel^91^. The astronauts exhibited a decrease in intrinsic connectivity in the right insula and ventral posterior cingulate cortex, which corresponded to a decrease in functional connectivity between the left cerebellum and brain regions associated with motor functions^92^. Following exposure to weightlessness in space, the expansion of CSF volume may exert pressure on the cerebellar parenchyma, potentially resulting in an elevation in the tortuosity of the ECS within the cerebellum^93^. Consequently, this could impede or halt the ISF drainage, thereby hindering the exchange between CSF and ISF. Both microglia activation and cerebellar neuron death contribute to the increase in tortuosity of ECS in the cerebellum^7^ (Fig. 6).

### Microgravity-related oxidative stress induces neuron death

Previous research has shown that microgravity influences the biological functions of mitochondria within cells, resulting in an increase in glycolysis, tricarboxylic acid cycle, reactive oxygen species levels, and NADPH oxidase activity^94^. However, oxidative phosphorylation and components of the mitochondrial respiratory chain are downregulated in this context^94^. Simulated microgravity induced oxidative stress in the cerebellar tissues of rats, resulting in a noteworthy elevation in reactive nitrogen species levels and a significant reduction in the overall antioxidant capacity within the cerebellar tissues after 21 days of tail suspension^95^. Following space flight, a decline in cytochrome oxidase activity was observed in rat cerebellar Purkinje cells, leading to an upsurge in free radical production and a decline in energy release, thereby impacting the proper functioning of the cerebellum^96^. Neuronal cells exhibited notable oxidative stress when subjected to simulated microgravity^97^. Meanwhile, neural stem cells may exhibit autophagy related events like caused by endoplasmic reticulum stress^98^. Notably, H_2_O_2_, the production of oxidative stress, has been found to induce FA generation^99^; subsequently, excessive FA induces the death of cerebellar neurons^7^. However, the damage to the cerebellum caused by microgravity is not persistent. It has been shown that the structural changes in the cerebellar cortex of rats were most significant on the 21st day of tail suspension, but showed a trend of recovery on the 28th day with adaptation^100^.

## Impairments of ECS and ISF by microgravity stress-derived fa

FA can quickly penetrate into cells and undergo chemical reactions with proteins or DNA molecules, forming intermolecular cross-linking or intramolecular chemical modifications^101^. By nucleophilic addition reactions with groups such as amino or imino groups in protein molecules, FA forms new covalent bonds between protein molecules and cross-links them^102^, thus altering the spatial structure of protein molecules. There have been studies in recent years suggesting that FA cross-links not only proteins within the cell, but also proteins in the ECS, leading to abnormalities in the ECS structure.

### FA-crosslinked ECM proteins in the brain ECS

One of the important structures for cells to sense gravity is the ECM in the ECS^103^, which includes components such as collagen, elastin, proteoglycans, aminoglycans, fibronectin, and laminin, while the expression of ECM proteins in the ECS can be affected by weightlessness conditions^104^. Surprisingly, weightlessness can increase the amount of ECM proteins and cytoskeleton in the papillary thyroid carcinoma cells^105^. Remarkably, excessive FA can fix brain tissues and lead to the hardening of tissue^106^. Especially, FA can crosslink ECM proteins^107^. Hence, FA-deposited ECM protein in the ECS is a direct factor of increasing the tortuosity of brain ECS and impeding ISF drainage.

### FA-crosslinked Aβ deposition in the brain ECS

**Fig. 7:**
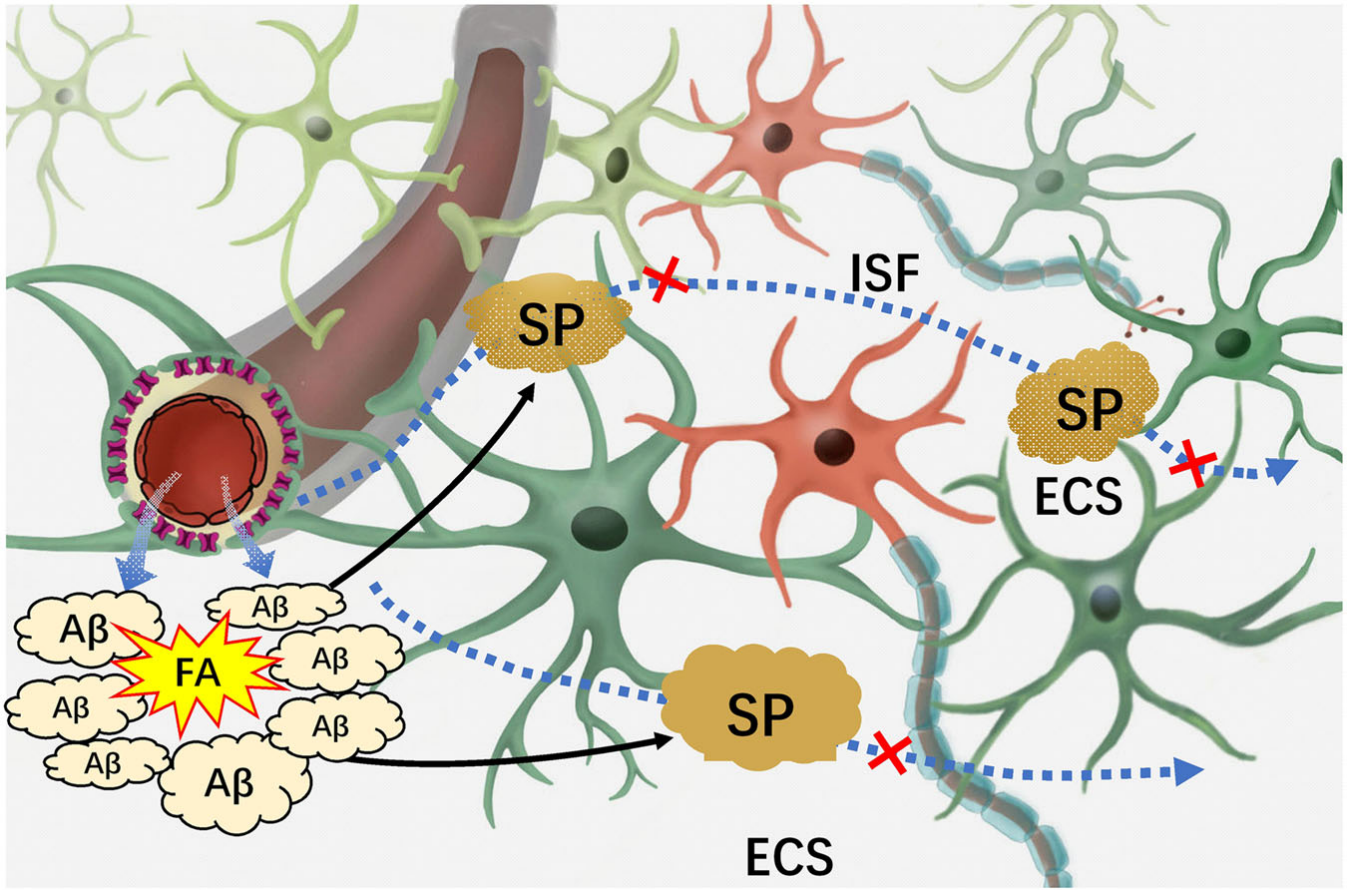
FA-crosslinked Aβ to form senile plaques and deposit in the brain ECS. In brief, FA can cross-link Aβ monomer to form AβO intracellularly and Aβ-related senile plaques in the brain ECS, which blocks brain ISF drainage and leading to neuron death. AβO Aβ oligomers, ECS extracellular space, FA formaldehyde, ISF interstitial fluid, SP senile plaques.

In the context of prolonged weightlessness, endogenous FA primarily originates from the oxidative deamination of methylamine catalyzed by SSAO. The presence of FA further intensifies intracellular oxidative stress^8^, and causes harm to vascular endothelial cells^108^. Additionally, FA readily binds to cysteine and lysine sites on proteins^109,110^, leading to the formation of cross-linking products between proteins and ultimately compromising the integrity of the ECS structure. Excessive FA have been observed to induce the aggregation of Aβ in vitro and in vivo by promoting the formation of Aβ oligomers and protofibrils^111^. This characteristic allows FA to cross-link harmless Aβ monomer depositions in the brain ECS, leading to the formation of Aβ-related senile plaques (SP). Recent study has found that sleep can scavenge Aβ from the brain to the peripheral blood; however, sleep disorders increase the risk of Aβ deposition in the brain^112^. Hence, Aβ-deposited in the ECS not only impairs the ECS structure but also impedes ISF drainage in the brain^6^, ultimately resulting in the demise of neurons located deep within the brain, such as the hippocampal neurons responsible for memory formation^59^. Under the condition of a disruption of the neuronal microenvironment, these neurons are unable to obtain adequate nourishment and effectively eliminate metabolic waste products (Fig. 7).

### FA-crosslinked albumin/hemoglobin deposition in ECS

**Fig. 8:**
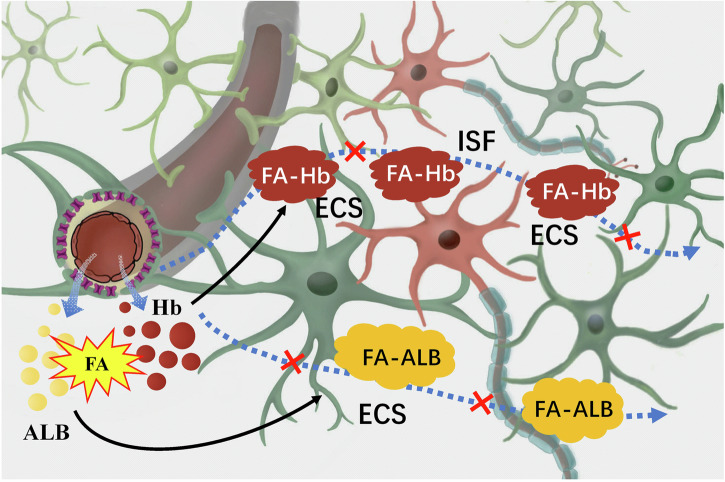
FA-crosslinked albumin/hemoglobin deposition in the brain ECS. In brief, albumin and hemoglobin infiltrated from blood vessels in the brain are cross-linked by FA to form macromolecules, which may block brain ISF drainage and lead to neuron death. ALB albumin, ECS extracellular space, FA formaldehyde, FA-ALB the complex of FA and albumin, FA-Hb the complex of FA and hemoglobin, Hb hemoglobin, ISF interstitial fluid.

Under physiological conditions, albumin contents in the plasma is much higher than that in the ISF; while hemoglobin is mainly present in the red blood cells of the plasma^113,114^. It is worth mentioning that the disruption of the blood-brain barrier (BBB) induced by microgravity in space triggers the penetration of albumin and hemoglobin from the blood vessels into the brain ECS^115^. When endothelial damage occurs in capillaries, resulting in increased vascular permeability, there is a notable influx of albumin into the brain ECS^116^. Meanwhile, some red blood cells in the blood will also enter the ECS through the vascular wall, leading to extravascular hemolysis and the release of a large amount of hemoglobin into the ECS^117^. Excessive FA has been found in the hippocampus and cerebellum after microgravity stress^7^. The interaction between albumin and FA induces structural changes in albumin, leading to a reduction in the quantity of α-helices^118^. This alteration results in a relaxation of the albumin framework and exposure of internal amino acids, potentially causing toxicity in organisms^118^. FA has been shown to possess the capacity to modify hemoglobin, generating a diverse combination of modified hemoglobin, including adducts with cross-linked hemoglobin chains^119^. In the presence of a disrupted BBB after space flight, FA cross-linked hemoglobin/albumin could accumulate in the brain ECS (Fig. 8).

Although cross-linked proteins can activate microglia to enhance their phagocytic and proteolytic activities^120^, excessive activation may lead to more protein cross-linking in brain ECS, causing a vicious cycle^121^. Cross-linked albumin/hemoglobin cannot break down under physiological conditions, blocking ISF drainage by taking up space in the ECS^109,110^.

### Role of miRNAs in regulating gene expression

**Fig. 9:**
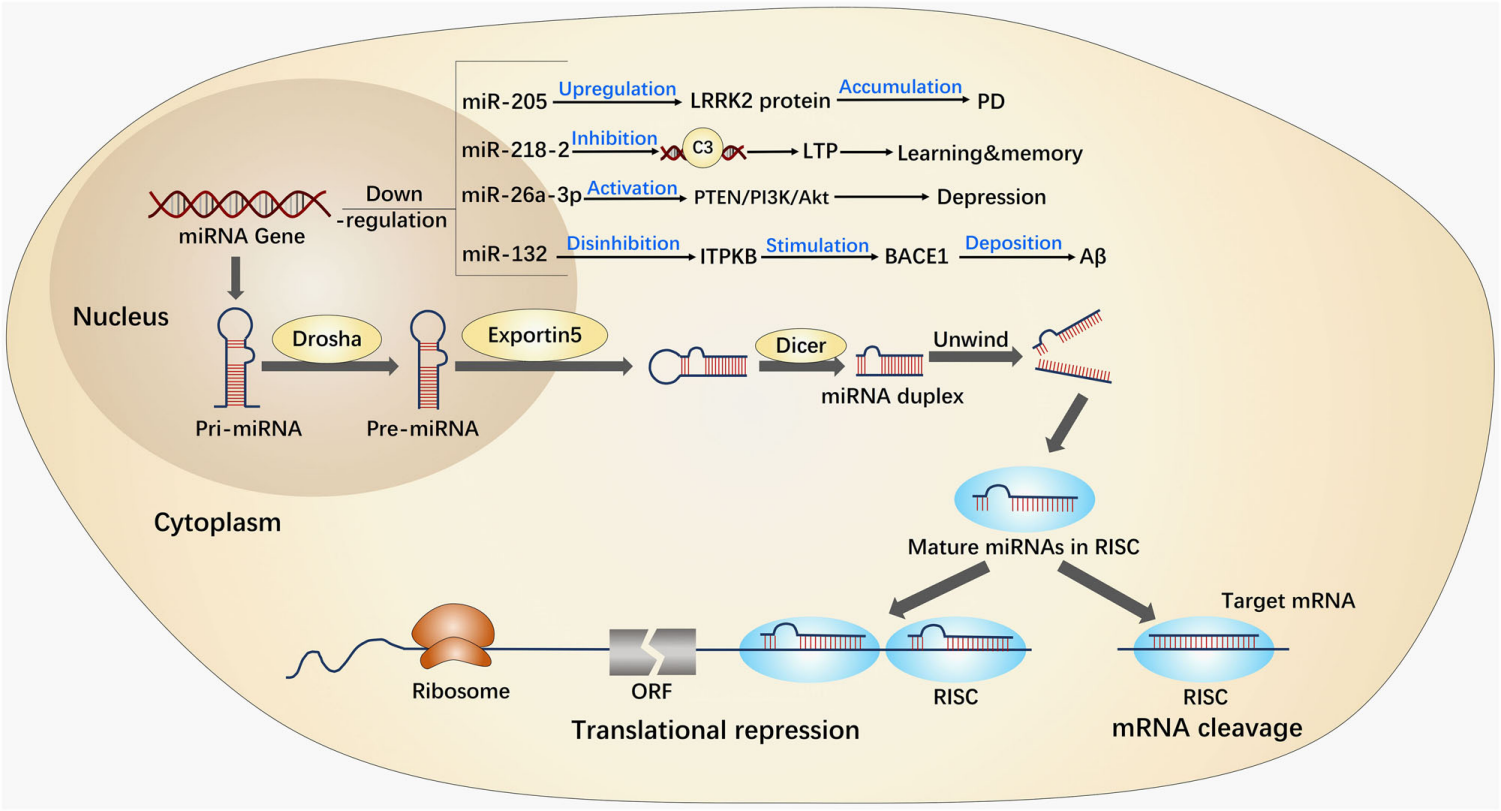
Schematic diagram of miRNA regulating gene expression and brain functions. In brief, miRNA exhibits diverse forms of existence, with the most rudimentary form being pri-miRNA, spanning ~300–1000 nucleotides. Following processing, pri-miRNA undergoes transformation into pre-miRNA, serving as the precursor to miRNA, measuring ~70–90 nucleotides. Subsequently, pre-miRNA is cleaved by the Dicer enzyme, ultimately yielding mature miRNA, which spans ~20–24 nucleotides. Then, miRNA possesses the capacity to direct RISC in the downregulation of gene expression at the post-transcriptional level, encompassing mRNA degradation or inhibition of mRNA translation. Hence, miRNAs exert regulatory control over brain functions implicated in neurodegenerative diseases. AD Alzheimer’s disease, LTP long-term potential, miRNAs microRNAs, ORF open reading frame, RISC RNA-induced silencing complex.

Multiple miRNAs have been identified in a wide range of organisms, including animals, plants, and viruses. Their discovery was serendipitous, occurring during a study on lin-14 in the nematode Hidradenitis elegans^122^. These miRNAs belong to a highly conserved class of endogenous non-coding small molecule single-stranded RNAs, which play a crucial role in post-transcriptional regulation of gene expression^123,124^. By binding to specific sites in the 3’ untranslated region of target genes and engaging in complementary base pairing, miRNAs can modulate the degradation or translation of target mRNAs^125^ (Fig. 9). Despite constituting a mere 2% of the overall human gene count, miRNAs are responsible for regulating at least 30% of human genes, with over 5300 human protein targets being subject to miRNA regulation by regulating different expression patterns^126–128^.

Numerous clinical studies have demonstrated that miRNAs play a crucial role in modulating central nervous system functions and are implicated in diverse biological processes^129,130^, including neuronal cell metabolism, proliferation, apoptosis, and the pathogenesis of various neurodegenerative disorders^129,130^. For example, the downregulation of miR-205 may contribute to an increase in the potential pathogenicity of LRRK2 protein in the brain of patients with sporadic PD^131^. Deficiency of miR-218-2 induces the deficits in the morphology and presynaptic neurotransmitter release in the hippocampus, impairing the abilities of learning and memory^132^. In addition, the deficiency of miR-26a-3p in the dentate gyrus of the hippocampus can activate the PTEN/PI3K/Akt signaling pathway, leading to neuronal deterioration and depressive- like behaviors^133^. The low levels of miR-132 upregulates ITPKB, leading to an increase in BACE1 activity and promoting the production of Aβ^134^ (Fig. 9).

Recent studies have revealed that FA has the capacity to modify the regulatory role of miRNAs in gene expression^15^. Specifically, FA has been found to disrupt the expression profiles of miRNAs in neuronal cells^16^, consequently leading to the dysregulation of certain miRNA expressions^17^, some of which may be implicated in microgravity-induced brain injury.

### Role of AQP4 in regulating ECS structure and ISF drainage

Aquaporin, a group of transmembrane proteins known for their water permeability, exhibits widespread expression on cell membranes and primarily functions in the regulation of intra- and extracellular water and electrolyte balance in diverse tissues and organs of organisms^135^. Among the 13 identified types of AQP in mammalian cell membranes, AQP1, AQP4, and AQP9 are particularly prevalent in brain tissues^136^. Recent studies have demonstrated that AQP4, a water channel protein, exhibits extensive distribution in the brain^137,138^. This protein not only plays a crucial role in the regulation of brain tissue water metabolism but also exerts significant influence on various processes such as CSF formation, glial lymphatic clearance, astrocyte activation and migration, and neural synaptic potential formation^139^.

AQP4 typically exhibits polarization and is distributed on astrocytes, forming channels primarily extending from the ends surrounding blood vessels to the entirety of the astrocyte membrane, as well as glial cells located around the ventricles and beneath the pia mater^140^. Concurrently, astrocytes assume a crucial role in facilitating the proper functioning of neural processes within the brain^141^. In addition to supplying nutrients to neuronal cells and mediating neurotransmitters, astrocytes also regulate the concentration of inorganic ions in the internal environment. In AQP4 knockout mice, the volume fraction of brain ECS is increased^142^, leading to a reduction in the clearance of waste products through the ISF^18^. Consequently, the dysregulation of the microenvironment results in neuron death.

### FA-regulated miRNA levels and AQP4 expression

Previous studies has demonstrated that FA has the potential to impair the expression profiles of miRNA in the neuronal cells^16^, thereby causing dysregulation in the certain miRNA expression, such as: miR-22, 26, 29, 125, 140, 142, 145, 203, 204, 320, 328, 344, 374, 485, 520, 1949, 3096, etc^15–17^. Especially, the expression of AQP4 could be regulated by these miRNAs including: miR-19a^143^, 29b^144^, 130a^145^, 130b^146^, 224^143^, 320a^147^, thereby perturbing the homeostasis of the brain’ ECS and ultimately culminating in neuronal demise. Notably, miRNA-29b, miRNA-145, and miRNA-320a have been identified as regulators of AQP4 expression within astrocytes^148^.

The upregulation of miRNA-29b has been found to enhance the expression of AQP4 in mouse brain tissue during cerebral ischemia, resulting in a reduction in the size of cerebral infarcts, mitigating the extent of edema, and minimizing disruption of the BBB^144^. Moreover, miRNA-29 in astrocytes plays a role in glutamate signaling and mitigates brain cell damage caused by excessive levels of glutamate^149^. In typical circumstances, the upregulation of miRNA-29b results in cerebral edema, thereby providing additional evidence of miRNA-29b’s mechanism of action through direct targeting of AQP4 expression^150^. In a rat astrocyte primary culture model subjected to oxygen and glucose deprivation, miRNA-145 mitigates the detrimental consequences of AQP4-induced astrocyte damage^151^.

Furthermore, in the context of cerebral edema, miRNA-320a exhibits a down-regulatory effect on the expression of AQP4, resulting in an elevation in cerebral infarct volume. Conversely, the utilization of an anti-miRNA-320a antibody to target miRNA-320a resulted in an upsurge in AQP4 expression and a reduction in infarct volume^147^. Previous studies have demonstrated that miRNA-320a can interact with the mRNA coding for AQP4, leading to inhibition of its translation or degradation, ultimately resulting in a downregulation of AQP4 expression^152^.

**Fig. 10:**
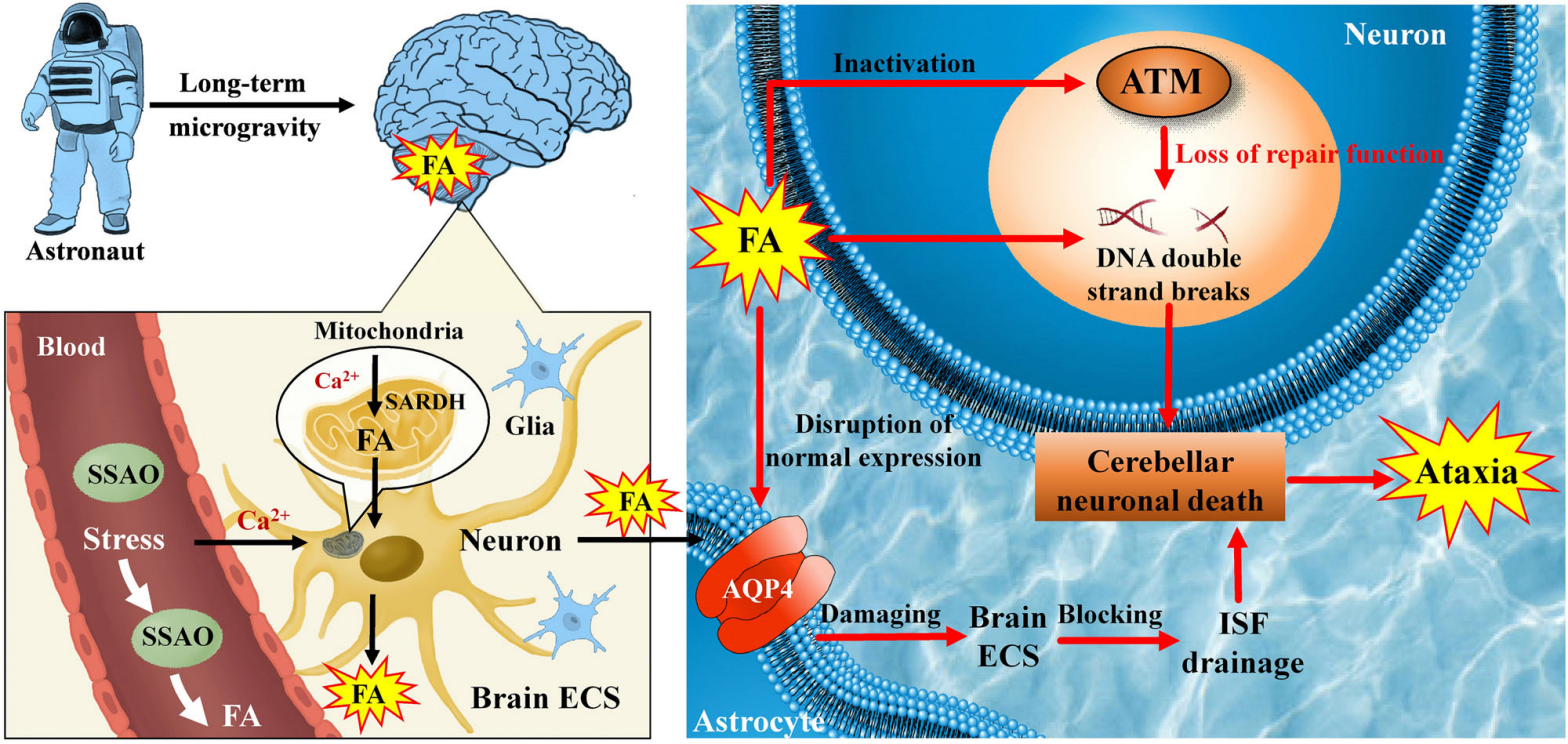
Roles of stress-derived FA in the cerebellum on microgravity-induced ataxia. In brief, during extended periods of space travel, microgravity can induce the activation of SSAO and SARDH enzymes, resulting in the production of FA within the cerebellum. This FA production can disrupt normal AQP4 expression within astrocytes, subsequently interfering with the ISF in the ECS. Consequently, this disruption in ISF drainage contributes to neuron death. Additionally, the accumulation of high FA may inactivate ATM protein kinase, thereby causing DNA damage in neurons and further promoting neuron death. These two factors seem to significantly contribute to ataxia onset. AQP4 aquaporin-4, ATM ataxia telangiectasia-mutated, CSF cerebrospinal fluid, ECS extracellular space, FA formaldehyde, ISF interstitial fluid, SARDH sarcosine dehydrogenase, SSAO semicarbazide-sensitive amine oxidase.

Interestingly, recent studies have found that AQP4 is upregulated in the foot of hippocampal astrocytes as a result of space flight^153^; the findings may differ in other brain regions, such as the cortex, where studies have demonstrated that there is no significant variation in AQP4 expression^153^. Weightlessness during spaceflight is highly likely to affect the levels of miR-29b, miR-145, and miR-320a due to the accumulation of FA in most of brain regions^148^, which subsequently causes disruption of normal AQP4 expression in the astrocytes. Hence, in certain regions of the brain, the decrease in AQP4 expression may hinder or slow down the ISF drainage of ECS in the brain, leading to neuronal death (Fig. 10). However, it is important to recognize that this phenomenon may not be occurring universally throughout the brain^148^.

## Roles of atm gene in ataxia occurence

The consensus in the scientific community is that cerebellar injury in humans results in the manifestation of ataxia, characterized by the organism’s inability to effectively regulate posture and balance, thereby leading to tremors and motor instability. Similarly, astronauts commonly experience these aforementioned symptoms upon reentry to Earth^154^. The ataxia telangiectasia-mutated (ATM) gene plays a crucial role in initiating DNA damage repair in cellular processes. It has been proven that ATM deficiency is strongly associated with ataxia^155^. This gene is located on the human chromosome 11q22-23 and encodes a protein kinase called ATM protein, which consists of 3056 amino acids^156^. The ATM protein kinase, belonging to the phosphatidylinositol 3-kinase (PI3K) family, primarily governs DNA double-strand breaks (DSBs) repair, cell cycle arrest, and apoptosis to uphold cellular genome stability and impede tumor initiation and progression^157^. Remarkably, FA can hinder DNA replication and cause DSBs in dividing cells^158^. Even when human cells are subjected to relatively low levels of FA toxicity, the presence of DSBs leads to an exceptionally robust and expeditious activation of the ATM pathway^159^, thereby initiating a sequence of intricate cascade reactions in downstream proteins. However, research has confirmed that the presence of DSBs is not the cause of the main wave of ATM activation by FA^159^.

The available evidence suggests that ATM protein kinases are frequently present as inactive dimers and are primarily located within the nucleus of higher eukaryotic tissue cells. However, their presence in the cytoplasm and nucleus of neurons remains largely consistent^160^. Numerous studies have demonstrated that mice lacking ATM protein kinase exhibit elevated levels of reactive oxygen species, particularly in the cerebellum, which is characterized by significant oxidative stress resulting in the degeneration and death of cerebellar neurons. Furthermore, activation of ATM protein kinase in the cytoplasm protects cerebellar neurons from damage due to oxidative stress^161–163^, which suggests that ATM protein kinase is closely related to the maintenance of cerebellar neurons’ vital activities. Especially, it has proven that inactivation of ATM protein kinase in cerebellar neurons causes symptoms of ataxia and movement disorders^164,165^. It has been proven that overexpression of miRNA-203 can downregulate the expression of the ATM gene^166^. Notably, exposure to FA can simultaneously downregulate the level of miRNA-203^17^, thereby reducing its inhibitory effect on ATM expression and enhancing the activation of ATM to repair DNA damage^166^. This may be one of the possible reasons why FA activates the ATM pathway (Fig. 10). Furthermore, the accumulation of low FA doses can cause strong and rapid activation of ATM signals in human cells^159^, which has a certain effect on protecting cerebellar neurons from damage. However, this study provides further evidence that the buildup of elevated levels of formaldehyde can deactivate ATM protein kinase through covalent dimerization and the creation of larger crosslinks^159^, at concentrations similar to those observed in the microgravity simulation model discussed earlier^7^. In summary, FA appears to be neuroprotective by upregulating the ATM gene at low levels, whereas at higher levels, it becomes toxic due to its ability to dimerize and inactivate ATM protein kinase.

## Conclusion and future perspectives

This review examines the effects of long-term space flight on astronaut health, specifically the impact of microgravity on memory function and motor ability, with endogenous FA accumulation playing a crucial role. The animal studies simulating microgravity mentioned in this review indicate that after prolonged exposure to microgravity, the concentration of FA in the hippocampus and cerebellum may abnormally increase^4,7^. This leads to pathological changes in these tissues similar to those seen in specific neurodegenerative diseases^59^, ultimately resulting in a decline in memory and motor function^7^. Moreover, microgravity-induced FA could influence miRNA expression, impacting the survival of nerve cells^15–17^. Hence, the regulation of FA metabolism has emerged as a promising target for drug treatment, offering novel insights and approaches for the advancement of therapeutic medications for memory and motor deficits associated with neurodegenerative disorders^111^.

To safeguard the enduring well-being of astronauts, global space centers have undertaken a sequence of drug development investigations pertaining to the amelioration of cerebral impairment induced by microgravity in space. Despite advancements in comprehending the molecular mechanisms that underlie the cognitive and motor impairments experienced by astronauts in weightless environments, the creation of efficacious pharmaceutical interventions remains elusive. This challenge arises from the presence of the BBB, which restricts the entry and functionality of conventional synthetic and protein-based drugs within the central nervous system^167^. However, the utilization of drug delivery mechanisms involving the lymphatic system and brain ECS presents a viable approach to circumvent the constraints imposed by the BBB, thereby offering a novel avenue for investigating therapeutic interventions targeting brain disorders. Nanopackaged coenzyme Q10 at 30 nm can penetrate both BBB and brain ECS (38–64 nm) to scavenge FA^111^, suggesting that nanomedicine may contribute to the improvement of astronaut health. In addition, due to its high stability in human fluids, miRNAs in the circulation can serve as biomarkers for the diagnosis and prognosis of astronaut diseases^168^. Thus, this review provides new insights into the role of FA in memory and motor impairments, which will help researchers design relevant drugs or other interventions to ensure the long-term health of astronauts in microgravity environment.
